# The association between mortality and use of Chinese herbal medicine among incident stage IV esophageal cancer patients: A retrospective cohort study with core herbs exploration

**DOI:** 10.3389/fphar.2022.1018281

**Published:** 2022-10-06

**Authors:** Shu-Ling Chen, Wei-Chun Lin, Yu-Chun Chen, Jiun-Liang Chen, Yi-Hong Wu, Sien-Hung Yang, Hsing-Yu Chen

**Affiliations:** ^1^ Division of Chinese Internal and Pediatric Medicine, Center for Traditional Chinese Medicine, Chang Gung Memorial Hospital, Taoyuan Branch, Taoyuan, Taiwan; ^2^ Department of Emergency Medicine, Chang Gung Memorial Hospital, Linkou Branch, Taoyuan, Taiwan; ^3^ Department of Family Medicine, Taipei Veterans General Hospital, Taipei, Taiwan; ^4^ School of Medicine, National Yang Ming Chiao Tung University, Taipei, Taiwan; ^5^ Institute of Hospital and Health Care Administration, National Yang-Ming University, Taipei, Taiwan; ^6^ School of Traditional Chinese Medicine, College of Medicine, Chang Gung University, Taoyuan, Taiwan; ^7^ Graduate Institute of Clinical Medical Sciences, College of Medicine, Chang Gung University, Taoyuan, Taiwan

**Keywords:** chinse herbal medicine, Chinese herbal medicine network, pharmacology network, stage IV esophageal cancer, survival analysis

## Abstract

Esophageal cancer (EC) remains a leading cause of death worldwide and in Taiwan. The prognosis of advanced-stage EC is notably poor, and the treatment options are limited. Chinese herbal medicine (CHM) has been widely used as a complementary treatment for cancer, yet the long-term effect of CHM in stage IV EC remains unclear.

The multi-institutional cohort obtained from the Chang Gung research database (CGRD) was used to study the long-term outcome of CHM use among incident stage IV EC patients from 1 January 2002, to 31 December 2018. All patients were followed up to 5 years or the occurrence of death. The overall survival (OS) and disease-specific survival rates were conducted using Kaplan-Meier estimation. Overlap weighing and landmark analysis were used to eliminate confounding and immortal time biases. Furthermore, we demonstrated the core CHMs for stage IV EC by using the Chinese herbal medicine network (CMN) analysis on prescriptions.

Nine hundred eighty-five stage IV EC patients were analyzed, including 74 CHM users and 911 non-CHM users. We found the use of CHM was associated with a higher 5-year overall survival rate than CHM nonusers (the cumulative probability: 19.52% versus 6.04%, log-rank test: *p* < 0.001, and the *p* < 0.001 with overlap weighting). In addition, the overall median survival time was about 7 months longer among CHM users. Moreover, the lower 1-, 3-, 5-year disease-specific survival rates were higher among CHM users. Additionally, the risk of all-cause mortality was lower among CHM users when considering accessible demographic covariates (adjusted hazard ratio: 0.59, 95%CI: 0.39, 0.89, *p* = 0.011). Furthermore, the CMN analysis revealed that CHMs improved health while relieving tumor burden. For example, *Hedyotis diffusa Willd* *.* was the core CHM with an anti-cancer effect, while *Fritillaria thunbergii* Miq and *Sevilla maindronide* Rochebrune were used together to relieve cancer-related gastrointestinal discomfort.

The use of CHM seems safe and possibly beneficial among stage IV EC patients with a higher 5-year OS. Further clinical trials on CHM were guaranteed to explore the role of CHM in managing stage IV EC patients.

## 1 Introduction

In recent years, esophageal cancer (EC) has remained one of the top ten leading causes of cancer death (Abnet et al., 2018). Even though the overall mortality rate has decreased recently, stage IV EC still has the worst prognosis among all EC patients. A 5-year relative survival rate of <8% globally is reported (Lu et al., 2010; Abnet et al., 2018; Cheng et al., 2018; He et al., 2020). For stage IV unresectable advanced or metastatic EC, treatment options are often limited and depend on each patient’s condition (Cheng et al., 2018; Kato et al., 2019; Kitagawa et al., 2019). Unfortunately, most patients in Taiwan were diagnosed at advanced stages (44.6% at stage III and 28.3% at stage IV) (Lai et al., 2020). Fluoropyrimidine plus platinum compounds are used as first-line therapy and taxanes as second-line therapy in stage IV EC patients. The median overall survival (mOS) could achieve 5.7 months (Cheng et al., 2018; Muro et al., 2019). Due to the complexity of stage IV EC, supportive care is also suggested throughout the management of stage IV EC. Multi-modalities may be needed for these advanced stage patients, including immunotherapy and HER2-targeted therapy (Kitagawa et al., 2019; Muro et al., 2019; Lu et al., 2021; Sun et al., 2021).

Chinese herbal medicine (CHM) has been widely used as a complementary therapy among several kinds of cancer patients. Chemotherapy-induced peripheral neuropathy, nausea, and vomiting are possible reasons to use CHM (Schröder et al., 2013; Liao et al., 2015; Hung et al., 2017; Chen et al., 2018; Song et al., 2019; Liao et al., 2020; Wong et al., 2021). Nonetheless, the role of CHM remains undetermined for stage IV EC since the information about the long-term prognosis of CHM users and the prescription patterns is still lacking. Most studies mentioned the feasibility of CHM in relieving discomforts without evaluating the survival benefits. Cui et al. reported that concurrent CHM use with chemoradiotherapy for treating mid and late-stage EC patients could lower the incidence and the severity of radiation-induced lung injury, provide better clinical benefits, and improve quality of life (QoL) (Cui et al., 2016). Moreover, Hu et al. (2011) reported that early infusion of CHM preparation *via* the enteral feeding tube after surgery for EC remarkably promoted the recovery of gastrointestinal function without causing additional abdominal symptoms and toxicities. On the other hand, survival benefits are often only reported among stage III or even earlier stage EC patients, and therefore the benefits other than symptom relief are unknown. A retrospective clinical study showed that chemoradiotherapy using a natural compound of CHM might improve stage II and III EC patients’ progression-free survival (PFS) and OS (Chang et al., 2017). After esophagectomy, CHM use was associated with a higher 3-year OS rate, superior QOL, and better immune function (Lu et al., 2006). Only one small-size study analyzed the CHM prescriptions made for EC patients in a real-world clinical database, which is vital to explore the commonly used CHMs for EC as the clinical reference (Cai et al., 2017). However, no cancer stage stratification was done in this study, and the prescriptions specialized for stage IV EC remain unknown.

This study aims to evaluate the potential role of CHM in managing patients with stage IV EC, and CHM prescription analysis is performed to disclose the core CHMs for stage IV EC. These results would be helpful in knowing the management strategy and feasibility of CHM among stage IV EC.

## 2 Materials and methods

### 2.1 Data source

The data of this study were retrieved from the Chang Gung Research Database (CGRD). The CGRD contains the original electronic medical records from the Chang Gung Memorial Hospital (CGMH), including gender, age, diagnosis of each outpatient/emergency visit or admission, medication, comorbidities, procedures, nursing cares, national health insurance payments, laboratory data, and cancer registry (Shao et al., 2019). The cancer registry database contained detailed information about diagnosis date, cancer stage, tumor size, dates of every treatment modality, date and type of recurrence, and date of death (Lee et al., 2021). CGMH is the largest private hospital system in Taiwan and comprises eight medical institutes: Keelung, Tucheng, Taipei, Linkou, Taoyuan, Yunlin, Chiayi, Kaohsiung, and Fengshan branches. CGMH covers about 20% of Taiwan’s population of cancer patients, while the outpatient coverage rates are up to 34%, which makes the CGRD a good target database for clinical study (Tsai et al., 2017).

CGRD also contains detailed CHM use records of CGMH patients, and all CHMs can be sorted into two groups: Single herb (SH) and herbal formula (HF). HF comprises SHs recorded in TCM classics, for example, Ban-Xia-Xie-Xin-Tang. On the other hand, SH represents a single material with the record in the CHM classics, for instance, *Hedyotis diffusa* Willd, or *Scutellaria barbata* D. Don. HFs are manufactured and mixed up with several SHs in a certain proportion according to the CHM classics before marketing. Hence TCM doctors may prescribe HFs and/or SHs accordingly based on every patient’s condition, and either HFs or SHs were treated as one item in the prescription, which means a prescription may contain more than one item. CHMs are all made by Good Manufacturing Practices pharmacies, and the potential renal or liver toxicities and pollution of pesticide or heavy metal are under zero-tolerance regulations.

### 2.2 Study population and ethical considerations

**FIGURE 1:**
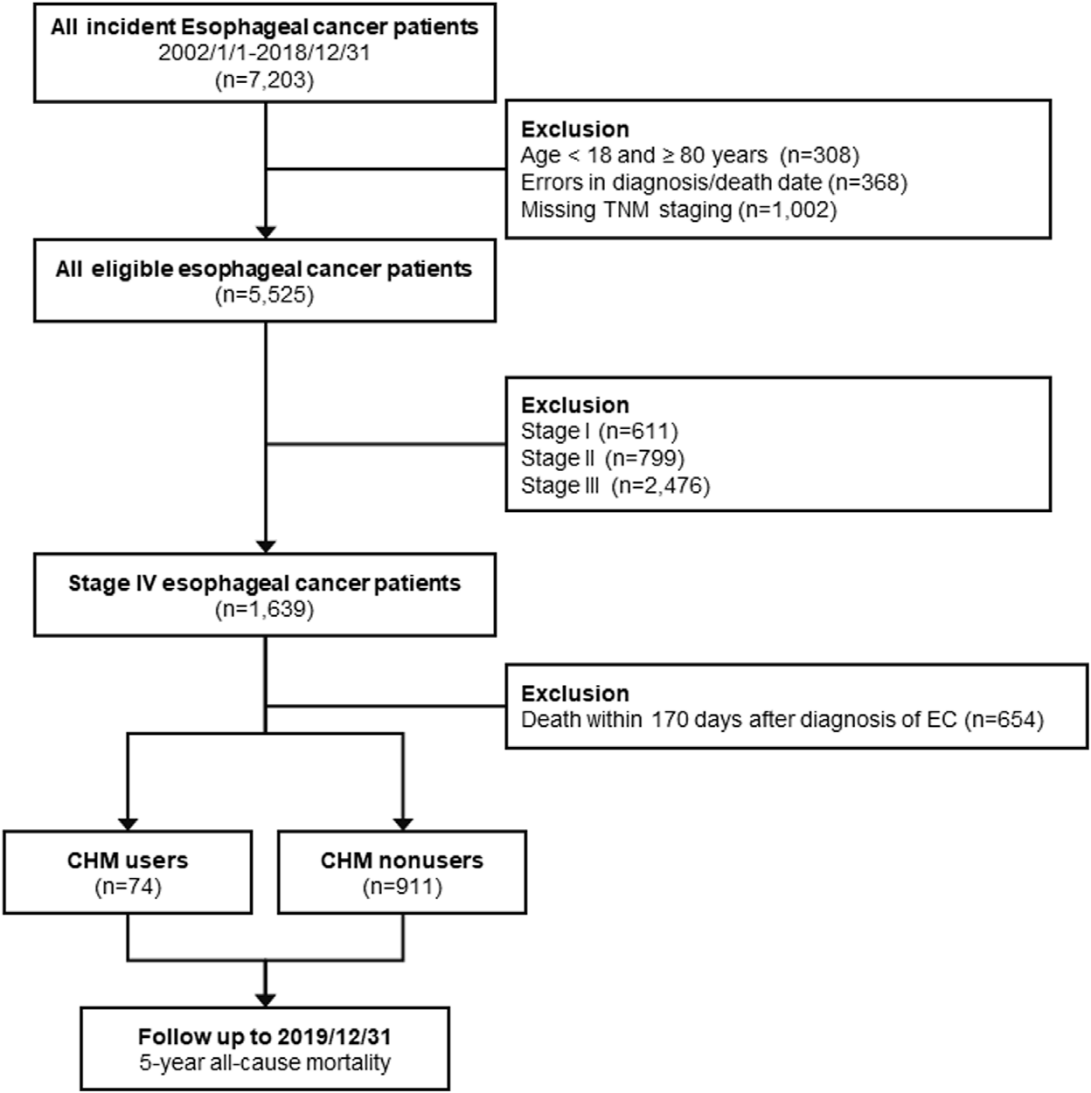
Flow chart of study design and selection procedure of study participants.

The International Classification of Diseases, 10th and Ninth Revision, Clinical Modification (ICD-9 and 10-CM) were used to determine the EC population. We included patients between 2002/01 and 2018/12 from CGRD who were newly diagnosed with stage IV EC (TNM staging, AJCC version 6 or 7; ICD-10-CM code: C15.0 -C16.2, ICD-9-CM code: 150). Patients with recurrence or relapsed EC from earlier stages were not included in this study. Furthermore, patients aged <18 years old or ≥80 years old, with errors in diagnosis or death date, missing TNM staging records, or those who had died within 170 days of diagnosis were excluded from this study. We classified the remaining eligible subjects into two groups based on whether CHM treatment was used after diagnosis. CHM users were defined as the population receiving at least one CHM treatment during the study period, and those who did not receive CHM treatment were classified as CHM nonusers. The intention-to-treat design was applied to define CHM users and nonusers, and therefore patients would not change to another group during follow-up. The study design is shown in the flowchart in Figure 1. The Institutional Review Board approved the entire study protocol of the Chang Gung Medical Foundation in Taiwan (IRB No.: 201900798B0C501). The written informed consents were waived because the identification number of each patient was well encrypted; hence the actual identity was impossible to be disclosed.

### 2.3 Outcome assessment

All eligible subjects were followed until the occurrence of the primary endpoint, 5-year at most after diagnosis, or the end of 2019 (Figure 1). The primary outcome of this study was OS, in which death could be caused by any cause, and the survival time was calculated from the date of diagnosis to the date of death. The secondary outcome of this study was disease-specific survival, in which only patients were died because of EC were enrolled in the survival analysis.

### 2.4 Study covariates

Demographic covariates, such as sex, age, body mass index (BMI), comorbidities, lifestyles, and pre-treat medications, were obtained from the CGRD. In addition, EC-related covariates were also acquired, including cancer staging, tumor size, and initial treatment modalities. Besides, biochemical profiles were obtained to present patients’ baseline physical condition, including the serum albumin, hemoglobin, platelet-to-lymphocyte ratio (PLR), neutrophil-to-lymphocyte ratio (NLR), and prognostic nutritional index (PNI) (Yodying et al., 2016; Pirozzolo et al., 2019; Okadome et al., 2020). The worst laboratory values were obtained within 1 year before diagnosis for the abovementioned biochemical profiles. In addition, two outpatient visits or one inpatient record of hypertension, type 2 diabetes mellitus (DM), myocardial infarction (MI), chronic obstructive pulmonary disease (COPD), cirrhosis, hepatitis B, hepatitis C, chronic kidney disease (CKD), and cerebrovascular diseases (CVD) within 1 year before the diagnosis of EC were considered as comorbidities. Also, Charlson Comorbidity Index (CCI) was used to summarize EC patients’ comorbidities and as an indicator associated with OS (Kim et al., 2020; Kubo et al., 2021). The diagnosis codes used in this study are listed in the Supplementary Table S1. Moreover, the use of metformin and aspirin were obtained since both may be related to EC survival (Honjo et al., 2014; Lin et al., 2020). As for initial treatments, which were rather complicated among stage IV EC patients, other than chemotherapy or radiotherapy, surgical interventions aiming at tumor reduction were investigated. Surgery types included local therapy and partial/total esophagectomy with or without gastrectomy. Additionally, pretreatment serum albumin, hemoglobin level, and BMI were acquired to assess the underlying nutrition status (Lim et al., 2017). Prognostic nutritional index (PNI) was used as a biomarker indicating both the patients’ nutritional status and systemic immune competence. Since PNI lower than 45 was highly associated with worse OS, hence we classified PNI groups into two groups (PNI≥45 and PNI<45) (Okadome et al., 2020). PLR and NLR as systemic inflammation and prognostic biomarkers of EC were also investigated (Yodying et al., 2016).

### 2.5 Bias assessment

To minimize the confounding bias, CHM users and nonusers were matched through different propensity score-based models as the sensitivity tests in this study. Furthermore, CGRD is electronic medical records updated simultaneously from daily clinical practices in the CGMH, which means that the possible recall bias could be ruled out (Sedgwick, 2012). In addition, immortal time bias may be concerned since there has been no exact timing of initiating CHM treatment for EC patients in the clinical guideline. Therefore, we excluded patients who died within 170 days during the follow-up period, which was determined by calculating the median time between the date of diagnosis and receiving CHM (Chuang et al., 2016). Furthermore, all the entries of CGRD have been linked to the national death registry database supported by the National Health Informatics Project. These data allowed us to trace patients’ outcomes afterward, even if the patient was deceased outside CGMH. Therefore, the possible registration and detection bias on death cause in CGRD could be eliminated (Tsai et al., 2017; Shao et al., 2021).

### 2.6 Statistical analysis

We conducted an outcomes assessment and the Chinese herbal medicine network (CMN) analysis on prescriptions made for EC in this study. We presented baseline demographic features as mean with standard deviation (SD) for continuous variables or count with percentage for categorical variables. We compared the differences between CHM users and nonusers with Student’s t-test and chi-square statistics. To balance CHM users’ and nonusers’ baseline status and to obliterate the imbalanced case numbers, we used propensity score (PS) with overlap weighting based on different demographic features between CHM users and nonusers: age, gender, comorbidities, BMI, and initial treatments (Li et al., 2018). The abovementioned covariates were used to generate the probability of using CHM as PS, and the PS was assigned to weight CHM nonusers while 1-PS was assigned to weight CHM users (Thomas et al., 2020). OS and disease-specific survival were estimated by the Kaplan-Meier method at 1-, 3-, 5-year. We calculated the hazard ratio (HR) of all-cause mortality with the Cox regression model and adjusted HR (aHR) by considering all accessible covariates other than covariates used to generate PS. Multivariate Cox regression stratified by demographic covariates and sensitivity tests with different models were also performed to confirm the associations between using CHM and the OS. We used PS-based models and different populations as sensitivity tests and subgroup analyses in this study. PS-based models included different PS-weight and matching methods, such as average treatment effect (ATE), the average treatment effect on the treated (ATT), overlap weighting, 1:1 propensity score matching (PSM) and kernel matching (Li et al., 2018). Models based on different populations included all subjects without landmark analysis, 90-day landmark analysis, subjects without any surgeries for tumor reduction, and populations with core CHMs acquired from CMN versus all CHM nonusers described as follows to describe effect of specific CHMs-based prescriptions.

Furthermore, core CHMs and possible pharmacologic mechanisms were explored by network pharmacology analysis. First, Chinese herbal medicine network (CMN) was used to graphically demonstrate the treatment principle and core CHM for EC. The build-up process of CMN was reported extensively in our previous studies. We used association rule mining to find out the common CHM combinations and social network analysis (SNA) to demonstrate and analyze the CMN graphically. We clustered commonly-used CHM according to the relations between CHMs and found core CHMs as the CHM with high prevalence and connections to other CHMs, which means that other CHMs were used when core CHM was prescribed (Chen et al., 2015; Guo et al., 2021; Wu et al., 2021). Stata (StataCorp. 2019. Stata Statistical Software: release 16. College Station, TX: StataCorp LLC) and NodeXLwere performed to analyze the CHN in this study to reveal the core CHMs among the rather complicated CMN, and *p* < 0.05 in the statistics was regarded as significant results.

## 3 Results

### 3.1 Baseline demographic features

From 2002/01/01 to 2018/12/31, 985 stage IV EC patients were analyzed in the final stage of our study, including 74 CHM users and 911 non-CHM users. Table 1 shows all analyzed subjects’ baseline demographic features, and no significant difference was noticed between groups in gender, age, lifestyles, tumor size, and biochemical profiles. Male patients were predominant (94.7% in CHM nonusers and 95.9% in CHM users) in both groups. The mean age of the subjects was 55.4 (10.1) years old in CHM users and 56.9 (9.4) years old in CHM nonusers, respectively, and most of the subjects in both groups fall between 41–60-year-old (64.1% of all eligible subjects). BMI was slightly higher among CHM users (22.3 versus 21.2 for CHM users and nonusers, respectively; *p* = 0.037). As for comorbidities, CHM users had about 5% higher rates of DM versus nonusers (*p* = 0.049), yet no significant differences were noted in hypertension, MI, COPD, CPD, CVD, PVD, HBV, HCV, liver cirrhosis, and CCI between the two groups. The medication use aligned with comorbidities analysis, in which CHM users had about 5% higher rates of metformin use (*p* = 0.024). Among stage IV EC patients, chemotherapy and radiotherapy were commonly used as the first treatment (61.0% and 51.5%, respectively), while only 7.6% of patients received surgery initially. Generally, more CHM users received initial therapies. The proportion of EC patients was 10–13% higher than CHM nonusers, whether for tumor-reduction surgery (16.2% versus 6.9%, *p* = 0.004 [data not shown]), chemotherapy (70.3% versus 60.3%, *p* = 0.09) and radiotherapy (63.5% versus 50.5%, *p* = 0.031).

**TABLE 1:** Baseline features of patients with stage IV esophageal cancer.

	All subjects (n = 985)	CHM nonusers (n = 911)	CHM users (n = 74)	*P*
Demographics				
Gender				
Female	51 (5.2%)	48 (5.3%)	3 (4.1%)	0.65
Male	934 (94.8%)	863 (94.7%)	71 (95.9%)	
Age (years)	55.5 (10.1)	55.4 (10.1)	56.9 (9.4)	0.22
Age group				
−40	59 (6.0%)	57 (6.3%)	2 (2.7%)	0.45
41–60	631 (64.1%)	583 (64.0%)	48 (64.9%)	
61-	295 (29.9%)	271 (29.7%)	24 (32.4%)	
BMI	21.3 (3.3)	21.2 (3.3)	22.3 (3.4)	0.037
Comorbidities				
DM	47 (4.8%)	40 (4.4%)	7 (9.5%)	0.049
Hypertension	115 (11.7%)	103 (11.3%)	12 (16.2%)	0.21
MI	5 (0.5%)	4 (0.4%)	1 (1.4%)	0.29
COPD	47 (4.8%)	44 (4.8%)	3 (4.1%)	0.76
CVD	20 (2.0%)	18 (2.0%)	2 (2.7%)	0.67
PVD	3 (0.3%)	3 (0.3%)	0 (0.0%)	0.62
Hepatitis B	32 (3.2%)	28 (3.1%)	4 (5.4%)	0.28
Hepatitis C	15 (1.5%)	15 (1.6%)	0 (0.0%)	0.27
Liver cirrhosis	63 (6.4%)	59 (6.5%)	4 (5.4%)	0.72
CKD	8 (0.8%)	7 (0.8%)	1 (1.4%)	0.59
CCI	9.2 (2.8)	9.2 (2.8)	9.4 (2.8)	0.51
Medications				
Metformin	63 (6.4%)	15 (1.6%)	4 (5.4%)	0.024
Aspirin	9.2 (2.8)	18 (2.0%)	3 (4.1%)	0.23
Lifestyles				
Cigarette smoking	264 (26.8%)	245 (26.9%)	19 (25.7%)	0.82
Alcohol consumption	250 (25.4%)	230 (25.2%)	20 (27.0%)	0.74
Betel nut chewing	117 (11.9%)	107 (11.7%)	10 (13.5%)	0.65
Tumor size (mm)	46.5 (39.0)	46.9 (39.2)	43.2 (37.1)	0.51
Initial treatment				
Surgery				
No surgery for tumor resection	910 (92.4%)	848 (93.1%)	62 (83.8%)	<0.001
Local therapy	1 (0.1%)	0 (0.0%)	1 (1.4%)	
Partial esophagectomy	14 (1.4%)	13 (1.4%)	1 (1.4%)	
Total esophagectomy	1 (0.1%)	1 (0.1%)	0 (0.0%)	
Esophagectomy with laryngectomy/gastrectomy	50 (5.1%)	41 (4.5%)	9 (12.2%)	
Other surgery	9 (0.9%)	8 (0.9%)	1 (1.4%)	
Chemotherapy	601 (61.0%)	549 (60.3%)	52 (70.3%)	0.090
Radiotherapy	507 (51.5%)	460 (50.5%)	47 (63.5%)	0.031
Biochemical profiles				
Albumin	3.8 (0.6)	3.8 (0.6)	3.9 (0.5)	0.13
Hemoglobin	12.9 (2.0)	12.9 (2.0)	13.3 (1.7)	0.14
ALT	22.5 (21.9)	22.6 (22.4)	20.9 (14.4)	0.55
PLR	183.0 (118.4)	183.4 (119.6)	177.4 (103.4)	0.70
NLR	4.3 (4.4)	4.3 (4.4)	4.0 (3.3)	0.52
PNI				
High (≥45)	409 (63.7%)	371 (63.0%)	38 (71.7%)	0.21
Low (<45)	233 (36.3%)	218 (37.0%)	15 (28.3%)	

Abbreviations: ALT, Alanine Aminotransferase; BMI, body mass index; CCI, Charlson Comorbidity Index; CHM, Chinese herbal medicine; CKD, chronic kidney disease; COPD, chronic obstructive pulmonary disease; CVD, cerebrovascular disease; DM, diabetes mellitus; MI, myocardial infarction; NLR, neutrophil-to-lymphocyte ratio; PLR, platelet-lymphocyte ratio; PNI, prognostic nutrition index; PVD, peripheral vascular disease.

### 3.2 Use of CHM associated with higher 5-year OS

**FIGURE 2:**
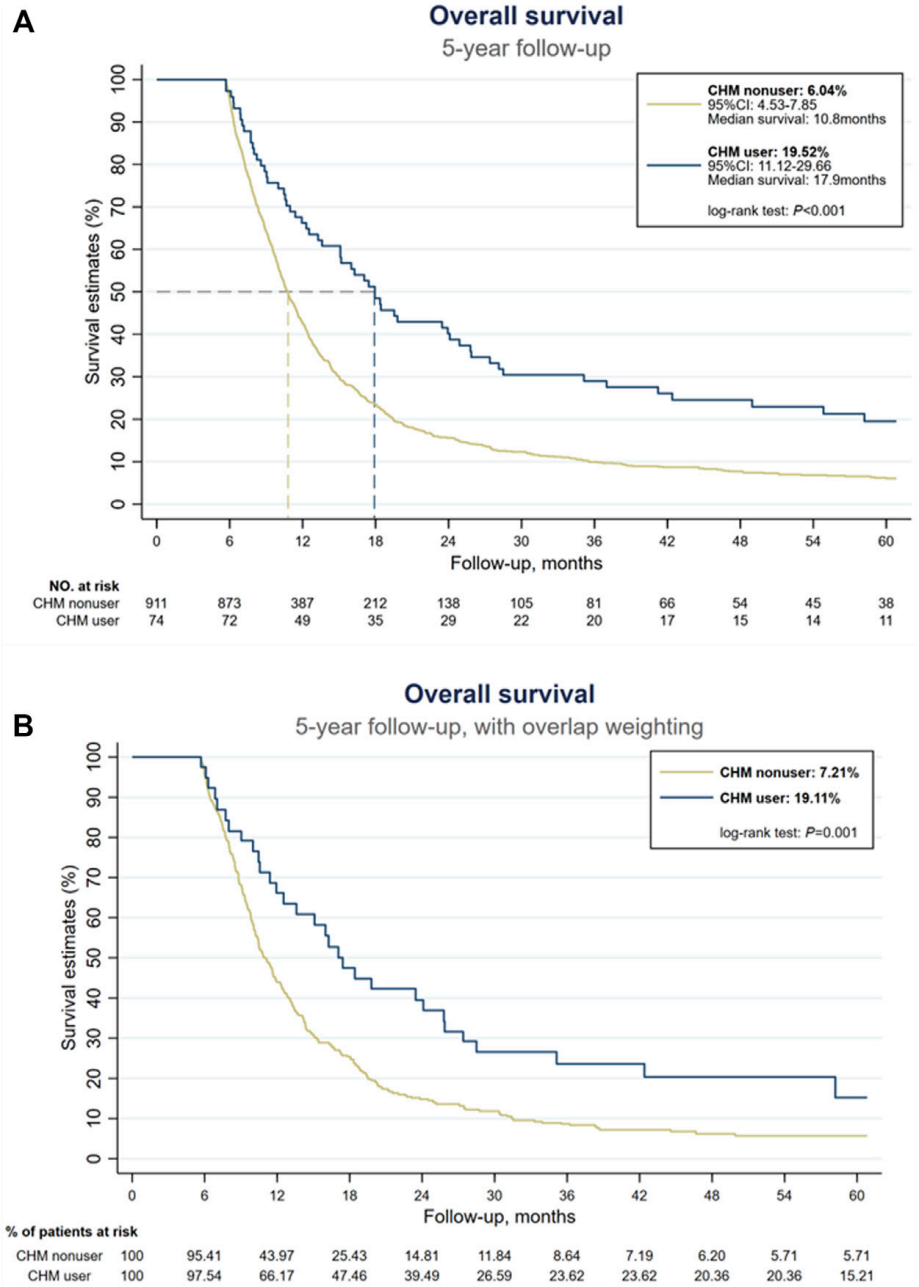
Kaplan-Meier estimates of overall survival of all stage IV esophageal cancer patients. **(A)** Without overlap weighting. **(B)** With overlap weighting.

At the end of the 5-year follow-up, 901 subjects were deceased among 985 subjects, of which 843 subjects were CHM nonusers (93.6% of all deceased subjects), and 58 subjects were CHM users (6.4% of all dead subjects). Under landmark design, whether with or without overlap weighting, the use of CHM was associated with a higher probability of 5-year OS than CHM nonusers (without overlap weighting: 19.52% versus 6.04%; with overlap weighting: 19.11% versus 7.21%, log-rank test: all *p* < 0.001, Figures 2A,B). Among CHM users, their mOS came to 17.9 months, while in CHM nonusers, their mOS was 10.8 months (Figure 2A). Table 2 shows the OS at 1-, 3-, and 5-year time frames, and the OS among all subjects shows a rapidly downward trend of OS. Further, CHM use was associated with higher OS at each time point than CHM nonusers. The favorable tendency for disease-specific survival could be seen when excluding subjects unrelated to EC at each time point (Table 2). Among CHM users, the risk of all-cause mortality could reduce by 46% compared with CHM nonusers (HR: 0.54, 95%CI: 0.37, 0.78, *p* < 0.001). When considering all accessible covariates, CHM users still had a 41% lower risk of all-cause mortality (aHR: 0.59, 95%CI: 0.39, 0.89, *p* = 0.011).

**TABLE 2:** The outcome of enrolled stage IV esophageal cancer patients.

	All subjects	CHM nonusers	CHM users	*P*
Overall-survival (OS) rate (%)				
1-year	44.4 (41.3, 47.5)	42.6 (39.4, 45.8)	66.2 (54.2, 75.8)	0.003
3-year	11.4 (9.5, 13.5)	10.0 (8.1, 12,0)	29.0 (19.1, 40.0)	<0.001
5-year	7.1 (5.5, 8.9)	6.0 (4.5, 7.9)	19.5 (11.1, 30.0)	<0.001
Disease-specific survival rate (%)				
1-year	44.3 (40.1, 47.8)	42.2 (38.5, 45.9)	66.2 (53.3, 76.2)	0.003
3-year	13.7 (11.3, 16.2)	11.8 (9.5, 14.4)	33.1 (22.0, 44.7)	<0.001
5-year	9.5 (7.4, 11.8)	8.3 (6.3, 10.7)	22.3 (12.7, 33.5)	<0.001

Abbreviations: CHM, Chinese herbal medicine.

### 3.3 Chinese herbal medicine network for EC patients

**FIGURE 3:**
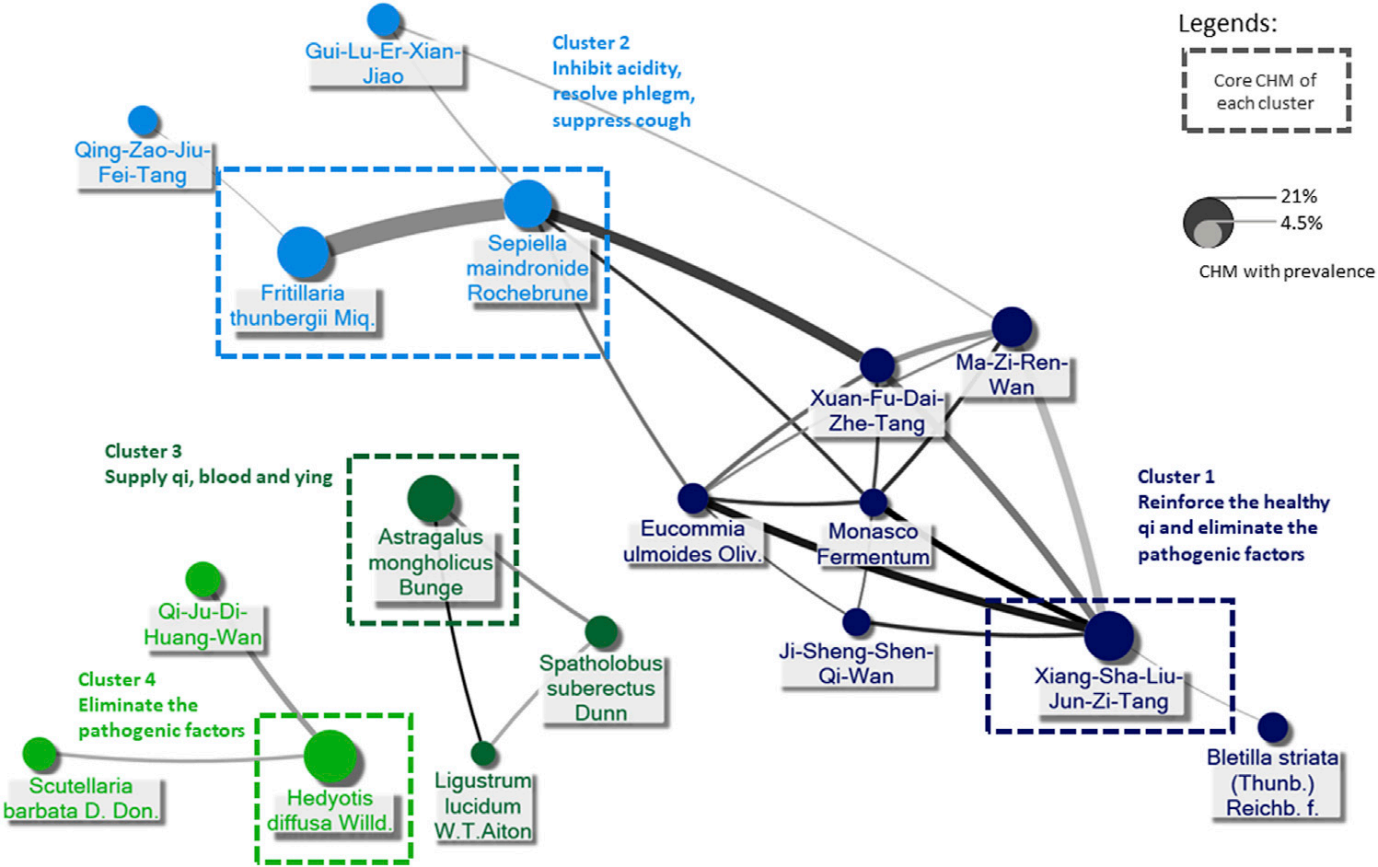
Demonstration of CHM prescriptions used for stage IV esophageal cancer (EC) by Chinese herbal medicine network (CMN). The core CHM of each cluster could be proposed by social network analysis (SNA). CHM indications were adopted from the Taiwan Herbal Pharmacopeia (4th edition, the Ministry of Health and Welfare, Taiwan), CHM indications provided by the website of the Ministry of Health and Welfare in Taiwan (https://dep.mohw.gov.tw/docmap/lp-874-108.html), and the Pharmacopeia of the People’s Republic of China, 2015 edition.

**FIGURE 4:**
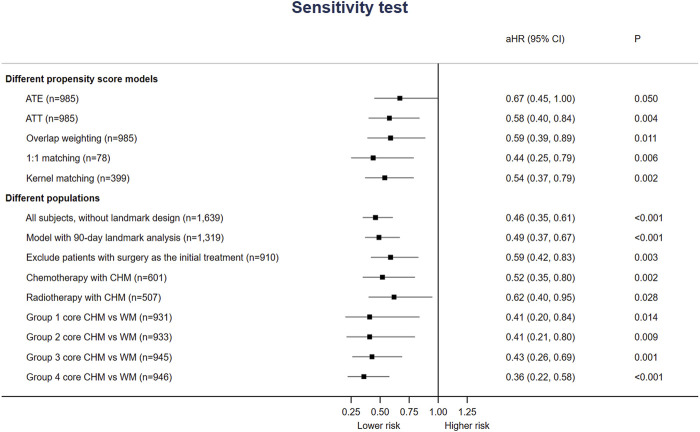
Sensitivity and subgroup analysis for overall survival estimation of CHM use.

Six hundred seventy-six prescriptions were made from 311 CHMs, and 6.9 kinds of CHMs were used in each prescription on average (SD: 2.7). Table 3, the list of the top ten prevalent single CHM, shows *Hedyotis diffusa Willd.* (21.2%) was the most used CHM for stage IV EC, followed by *Fritillaria thunbergii* Miq. (20.0%) and Xiag-Sha-Liu-Jun-Zi-Tang (XSLJZT, 18.9%). Furthermore, the top 30 prevalent CHM-CHM combinations were used to construct CMN, and SNA revealed the core CHMs (Figure 4). Table 4 shows the top ten commonly used CHM combinations as an example and the top 30 prevalent CHM combinations used for CMN (Supplementary Table S2). After clustering, the CHMs of each cluster are listed in Supplementary Table S3, and the composition of HF in the network is listed in Supplementary Table S4. CHM with larger circles meant higher prevalence in the CMN, thicker connecting lines represented higher prescription frequency, and darker connection lines indicated closer relations between connected CHMs. These CHMs could be clustered to 4 groups according to their connections, and the core CHMs among the four clusters could be found due to the relatively high prevalence and more connections to other CHM within clusters (Chen et al., 2015; Wu et al., 2021). For example, XSLJZT was the core CHM in group 1 to relieve the negative impact on gastrointestinal tract; while *Fritillaria thunbergii* Miq with *Sepiella maindronide* Rochebrune were the core CHMs in group 2 to ease cough and synergistically enhance the effect on gastrointestinal tract. *Astragalus mongholicus* Bunge. Was the core CHM in group 3 to relieve cancer-related fatigue, and *Hedyotis diffusa Willd.* Was the core CHM in cluster 4 with an anti-cancer effect. By integrating CHM indications from CHM pharmacopeia into clustered CMN, we could find out the CHM features of each cluster, including reinforcing the healthy qi and eliminating the pathogenic factors (group 1), inhibiting acidity, resolving phlegm, suppressing cough (group 2), supplying qi, blood, and yin (group 3), and eliminating the pathologic factors (group 4) (Figure 3). The core CHM is also associated with lower all-cause mortality risks than CHM nonusers (Figure 4).

**TABLE 3:** The top five herbal formula (HF) and single herb (SH) prescribed for stage IV esophageal cancer (prescription number: 676).

CHM	CHM type	Counts	Prevalence (%)
Herbal formula (HF)			
Xiang-Sha-Liu-Jun-Zi-Tang	HF	128	18.9
Ban-Xia-Xie-Xin-Tang	HF	107	15.8
Ma-Zi-Ren-Wan	HF	84	12.4
Xuan-Fu-Dai-Zhe-Tang	HF	63	9.3
Qi-Ju-Di-Huang-Wan	HF	63	9.3
Single herb (SH)			
*Hedyotis diffusa* Willd	SH	143	21.2
*Fritillaria thunbergii* Miq	SH	135	20.0
*Sepiella maindronide* Rochebrune	SH	119	17.6
*Astragalus mongholicus* Bunge	SH	114	16.9
*Salvia miltiorrhiza* Bunge	SH	64	9.5

Abbreviations: CHM, Chinese herbal medicine; SH, Single herb; HF, Herbal formula.

**TABLE 4:** The top ten two CHM combinations for stage IV esophageal cancer. Prevalence was the percentage of each combination of all prescriptions (n = 676). Confidence and lift presented the strength of each combination, and a higher value represented stronger connections between CHMs.

CHM a	CHM B	Prevalence (%)	Confidence	Lift
*Fritillaria thunbergii* Miq	*Sepiella maindronide* Rochebrune	10.9	62.2	3.11
*Sepiella maindronide* Rochebrune	Xiang-Sha-Liu-Jun-Zi-Tang	7.7	40.6	2.31
Xuan-Fu-Dai-Zhe-Tang	*Sepiella maindronide* Rochebrune	7.4	42.0	4.51
*Hedyotis diffusa* Willd	*Astragalus mongholicus* Bunge	6.7	39.5	1.87
Xiang-Sha-Liu-Jun-Zi-Tang	Ma-Zi-Ren-Wan	6.7	53.6	2.83
*Eucommia ulmoides* Oliv	Xiang-Sha-Liu-Jun-Zi-Tang	6.4	33.6	4.73
Xuan-Fu-Dai-Zhe-Tang	Xiang-Sha-Liu-Jun-Zi-Tang	6.2	32.8	3.52
*Hedyotis diffusa* Willd	Ban-Xia-Xie-Xin-Tang	6.1	38.3	1.81
Monasco Fermentum	Xiang-Sha-Liu-Jun-Zi-Tang	5.8	30.5	5.15
Xuan-Fu-Dai-Zhe-Tang	Ma-Zi-Ren-Wan	5.6	45.2	4.85

Abbreviations: CHM, Chinese herbal medicine.

### 3.4 Sensitivity and subgroup analysis

Additionally, Figure 4 shows that the association between CHM use and lower risk of all-cause mortality was consistent among different models or sampled populations among all stage IV EC patients in the sensitivity/subgroup tests. Whether including patients with surgery for EC as the initial treatment of surgery or not, the risk of all-cause mortality among CHM users was still lower (aHR 0.59, 95%CI: 0.42–0.83, *p* = 0.003). Since surgery could be applied to quite small proportion of incident stage IV EC patients, we add analysis on the survival benefit of patients with chemotherapy and radiotherapy. The aHR was 0.52 (95%CI: 0.35, 0.80) and 0.62 (95%CI: 0.40, 0.95) for patients received chemotherapy and radiotherapy, respectively. The benefit seemed still consistent with over populations or PS models.

## 4 Discussion

This is the first study evaluating the long-term prognosis of stage IV EC patients using CHM and graphically demonstrating the CMN with the core CHM for EC patients. Our results support the long-term use of CHM since that CHM users had higher 1-, 3- and 5-year OS and disease-specific survival rates (Table 2 and Figure 2). Also, the risk of all-cause mortality was significantly lower with or without considering all accessible covariates in the regression model (Figure 4). This study provides that CHM may be beneficial for prolonging stage IV EC patient’s life, which could be a crucial reference and supplement to previous studies about symptom relief only (Yang et al., 2012; Yeh et al., 2014; Chen et al., 2016; Hsu et al., 2016). We also performed sensitivity tests with different PS-based matching methods (ATE, ATT, overlap weighting, and kernel matching) and other populations (with 90-day landmark analysis, without excluding patients who died within 170 days after the diagnosis, without patients having surgery as initial treatment) to avoid potential confounding and selection biases. Since CHM is not yet a part of the standard supportive management of stage IV EC, the consistent results of ATE and even lower HRs in ATT models show the feasibility of using CHM among stage IV EC patients.

The rate of OS shown in this study is comparable to previous studies, and this result suggests the reliability of using CGRD as the study material (Cheng et al., 2018; Akhtar-Danesh et al., 2019; He et al., 2020; Jung et al., 2020; Watanabe et al., 2021). Also, since guidelines recommend management based on multiple modalities for EC, the improvement in OS among CHM users provides the potential for using CHM as part of the management modalities for stage IV EC patients (Kitagawa et al., 2019; Muro et al., 2019). Akhtar-Danesh et al. (2019) reported that the 5-year OS rate of stage IV EC patients was 0.8% with chemotherapy or radiotherapy alone, 5% with chemoradiotherapy, and 19.7% with surgical resection. A much better 5-year OS rate of stage IV EC was reported in Japan: 17.2% with definitive chemoradiotherapy and12.9% with radiotherapy alone (Watanabe et al., 2021). A Korean multi-institutional cohort study showed similar data that with multiple treatment modalities, the 5-year OS rate was about 16.6%, and mOS was 0.9 years (Jung et al., 2020). In China, a retrospective controlled study reported that the 3-year OS rate of advanced-stage EC patients was about 4.7%–10.7%, and the mOS was about 11–14 months (Lyu et al., 2018). In our study, even though the 5-year OS seemed similar among all eligible subjects, the higher OS rate among CHM users revealed the possibility of integrating CHM with palliative management. Additionally, in our study, CHM users’ 5-year OS rate was 19.52%, and their mOS was 17.9 months, roughly 4 months longer than EC patients with positive PD-L1 (mOS: 13.9 months) from KEYNOTE-590 (Sun et al., 2021). Although clinical studies about the use of CHM and immunotherapy for stage IV EC are still lacking, our results imply that CHM may be used if immunotherapy is not available; for example, tumors without PD-L1 expression unaffordable price of immunotherapy, or the window before starting immunotherapy.

As for surgical intervention, surgery is recommended for all operable EC patients, either followed by concurrent chemo-radiation therapy (CCRT) and chemotherapy/radiation therapy alone or not. It may be a positive factor for CHM users since the use of CHM was associated with a higher proportion of surgical intervention. Although the role of esophagectomy has not been well established in the palliative management of advanced-stage EC, the survival could be better in patients undergoing palliative esophagectomy with specific conditions, such as single-organ oligometastatic disease (Depypere et al., 2018). Another recent study suggests that only selected subsets of patients with primarily non-visceral, non-osseous metastatic EC have favorable survival and may benefit from aggressive local therapies (Seyedin et al., 2021). However, metastasis and unresectable tumors are common for stage IV EC patients, and surgery may not suit most cases. More than 90% of patients did not receive an operation for the tumor in our study, and therefore, the influence of surgery on the outcome assessment should be minimal. Also, the consistent results in different PS models and Cox regressions model may support the use of CHM as an independent factor for a better outcome.

The CHM prescriptions for stage IV EC seemed quite diverse due to the complexity of disease presentation, which was why CMN analysis was crucial for this study. Based on TCM theory, the CHM prescriptions may be made according to the individual condition, and we could identify 4 clusters of CHM with different indications according to the connections between CHMs. Our CMN results show the most prescribed SH, *Hedyotis diffusa* Willd., was the core CHM of the cluster to eliminate the pathogenic factors of EC, and it was found to have anti-cancer, antioxidant, and anti-inflammatory effects (Chen et al., 2008; Ye and Huang, 2012; Tao and Balunas, 2016). Furthermore, Li et al. reported that the active ingredients of Hedyotis Diffusa Willd, 1,3-dihydroxy-2-methyl anthraquinone, and the ethyl acetate fraction showed potential anti-cancer effects, and the mechanisms of action may involve mitochondrial apoptotic and death receptor pathways (Li et al., 2016).

The fact that up to 79% of EC patients developed gastrointestinal discomfort, malnutrition, and loss of body weight is present in more than 70% of EC patients at the time of diagnosis may explain why the second most common CHM combination is *Fritillaria thunbergii* Miq and *Sepiella maindronide* Rochebrune (Larrea et al., 1992; Anandavadivelan and Lagergren, 2016; Nam et al., 2020). They were also the core CHM of the cluster that inhibits acidity, resolves phlegm, and suppress cough. Among EC patients, dysphagia and esophagus dysmotility or dyskinesia are vital symptoms used to diagnose the stomach and liver disharmony by TCM clinicians. Many effective compounds of *Fritillaria thunbergii* Miq were recognized. Still, Peimine, the main ingredient of *Fritillaria thunbergii* Miq was found to have expectorant, antitussive, anti-inflammation, and anti-ulcer properties; hence was widely applied in the airway and gastric disease (Kim et al., 2016; Nile et al., 2021). The other most used HF, XSLJZT, following *Fritillaria thunbergii* Miq and *Sepiella maindronide* Rochebrune, was also commonly used and studied as Japanese Kampo, known as Rikkunshito. Since cancer anorexia and cachexia involve decreased ghrelin signaling due to excessive hypothalamic interactions of 5-HT with corticotropin-releasing factor through the 5-HT2cR (Fujitsuka et al., 2009), reported that the active components of XSLJZT, hesperidin and atractylodin, potentiate ghrelin secretion and receptor signaling, respectively, and atractylodin prolonged survival in tumor-bearing rats (Fujitsuka et al., 2011; Fujitsuka and Uezono, 2014). Furthermore, XSLJZT was also reported to ameliorate anorexia, GI dysmotility, muscle wasting, and anxiety-related behavior and prolonged survival in animals and patients with cancer (Suzuki et al., 2014; Tominaga et al., 2018). The connections between clusters 1 and 2, or precisely core CHMs within cluster 1 and cluster 2, indicated the CHM’s role in complementary care by caring for patients’ discomfort while fighting against the tumor itself at the same time.

Asides from the CHMs eliminating the pathogenic factors and modulating gastrointestinal discomfort, there are also other CHM in the CMN that was proved to have an antitumor effect and are involved in immune regulation, like the idea of immunotherapy. For example, the core CHM of supplying qi, blood, and yin, *Astragalus mongholicus* Bunge, was reported that its main component, *Astragalus polysaccharide* (PG2) modulates the M1/M2 macrophage pool, facilitates dendritic cells maturation and synergistically enhancing the anticancer effect of platinum compounds. (Bamodu et al., 2019). Another SH from the same cluster, *Spatholobus suberectus* Dunn, was reported that (-)-Sativan, a naturally isolated isoflavone from *Spatholobus suberectus* Dunn, could inhibit PD-L1 expression and epithelial-to-mesenchymal transition by up-regulating miR-200c in triple-negative breast cancer cells (Peng et al., 2020).

Still, this study has several limitations. First, CGRD covers only CHM prescribed from CGMH. The EC patients who received CHM or other therapies at local clinics or other medical facilities could not be identified; hence the CHM use may be underestimated. Second, for late-stage EC patients, dysphagia may be severe and influence the nutrition status and even prognosis, which may cause potential selection bias and overestimate the effect size among CHM users. In practice, enteral feeding was the first option for EC patients for nutrition support instead of the parental route, and CHM treatment works well with every type of enteral feeding (Bozzetti, 2010). Since most EC patients would have an enteral feeding gateway established, and the insignificant differences in pretreatment serum albumin, hemoglobin level, and PNI between CHM users and nonusers. The bias could be minimized. Third, since CHM is not a standard treatment for EC, there’s no suggestive timing for initiating CHM management. Patients themselves often ask for CHM management from TCM doctors according to their demands in clinical practice. Therefore, accidental exposure to CHM among CHM nonusers should be minimal, and the use of landmark analysis could lower the possibility of immortal time bias. Forth, the proportion of CHM users were low in this study. Currently, the information about prevalence of CHM use among EC patients remains unclear, and the low proportion of CHM use may be caused by the fact that we only enrolled the incident stage IV EC patients and the difficulty in using CHM through enteral or oral feeding. However, the low proportion of CHM use among incident stage IV EC may still raise the issue of the generalizability of this study. Similarly, the nature of male predominance in EC may cause inference errors when applying the results to female patients. Studies based on more hospitals are demanded to confirm the role of CHM in managing stage IV EC as external validation. Finally, our study was still a retrospective observational study and limited by the database, so further large randomized controlled trials with specific SH or HF of CHM are still needed to confirm the actual causality of different prescriptions.

## 5 Conclusion

Our study showed that CHM use may be associated with a better outcome among stage IV EC patients and could be safe when used as a part of supportive care. Besides, the core CHM and CHM combinations explored by the CMN disclosed that eliminating pathogenic factors and relieving gastrointestinal discomforts were important CHM indications. These results may warrant further clinical and bench studies for the advanced stage of EC.

## Data Availability

The original contributions presented in the study are included in the article/Supplementary Material, further inquiries can be directed to the corresponding author.
