# Supplementary material for: The association between mortality and use of Chinese herbal medicine among incident stage IV esophageal cancer patients: A retrospective cohort study with core herbs exploration
Source: Front Pharmacol. 2022 Oct 6;13:1018281. doi: 10.3389/fphar.2022.1018281 (PMC9582778; doi:10.3389/fphar.2022.1018281)
Supplement: Supplementary file 3 [file Table1.DOCX]

**Supplementary Table S1.** Diagnosis codes used in the study

| Disease | ICD-9-CM codes | ICD-10-CM codes |
| --- | --- | --- |
| Hypertension | 401-405 | I10, I11 |
| Type 2 DM | 250.x, except 250.x1 and 250.x3 | E11 |
| MI | 410, 411, 412 | I21-I22, I252 |
| COPD |  | J42-J44 |
| Hepatitis B | 07020-07023, 07030-70733 | B169, B1910, B162, B1911, B181 |
| Hepatitis C | 07041, 07044, 07051, 07054 | B1710, B1711, B182, B1920, B1921 |
| Liver cirrhosis | 5712, 5715, 5716 | K74 |
| CKD | 580.x-588.x, 250.4x, 274.1x, 283.11, 403.x1, 404.x2, 404.x3, 440.1, 442.1, 447.3, 572.4, 642.1x, 646.2x and 791.0 | N032-N037, N052-N057, N18, N19 |
| CVD | 430-438 | I60-I69 |

Abbreviations: CAD, coronary artery disease; CKD, chronic kidney disease; CVA, cerebrovascular accident; HF, heart failure; ICD-9-CM, International Classification of Diseases, 9^th^ Revision, Clinical Modification
